# Pharmacological inhibition of RAS pathway alleviates spine deformity in a mouse model of neurofibromatosis type 1

**DOI:** 10.1038/s41413-025-00492-3

**Published:** 2025-12-16

**Authors:** Franceska Kovaci, Cassandre Goachet, Simon Perrin, Lotfi Slimani, Fanny Coulpier, Françoise Tilotta, Piotr Topilko, Céline Colnot

**Affiliations:** 1 Univ Paris Est Creteil, INSERM, IMRB, Creteil, France; 2 Université Paris Cité, Inserm UMR_S 1333 Oral Health, Montrouge, France; 3 Department of Oral Medicine, AP-HP Paris Saclay – Sainte-Périne Hospital, Paris, France

**Keywords:** Bone, Pathogenesis

## Abstract

Neurofibromatosis type 1 (NF1) is a genetic disorder affecting 1 in 3 000 people due to heterozygous mutations in the *NF1* gene. Patients with NF1 can develop multiple symptoms, such as neurofibromas, skin hyperpigmentation, and bone abnormalities, including tibial pseudarthrosis and spine deformity. Here, we aimed to elucidate the cellular origin and pathogenic mechanism of NF1 spine deformity. We explored the *Prss56-Nf1* knockout (KO) mouse model that recapitulates neurofibromas and pseudarthrosis by carrying *Nf1* gene inactivation in Prss56-expressing boundary cap cells, a neural crest subset, and their derivatives. Micro-CT analyses showed that *Prss56-Nf1* KO mice exhibit spine deformity from 12 months of age, associated with vertebral anomalies reminiscent of patients with NF1. Fate mapping revealed a significant increase in OSX^+^ osteoblasts of the Prss56 lineage in vertebrae of *Prss56-Nf1* KO mice. Increased traced *Nf1*-deficient cells correlated with increased vertebral bone volume and kyphosis spine curvature. Finally, we showed that treating *Prss56-Nf1* KO mice with RAS-MAPK pathway inhibitors prevented spine deformity. Overall, the *Prss56-Nf1* KO mouse model unravels the role of osteoblasts from the Prss56 lineage as the cellular origin of NF1 spine deformity and highlights RAS-MAPK pathway inhibition as a promising therapeutic strategy for preventing NF1 spine deformity.

## Introduction

Spine deformities, including scoliosis and kyphosis, are characterized by abnormal curvature of the vertebral column that can lead to pain, reduced mobility, and, in severe cases, impaired organ function and morbidity.^1–3^ Scoliosis is classified as congenital, neuromuscular, syndromic, or in most cases “idiopathic” affecting primarily adolescents.^4–7^ The prevalence of early onset scoliosis is 3%-4.5% in the general population but increases to 10%-69% in neurofibromatosis type 1 patients.^8–12^ Neurofibromatosis type 1 (NF1) is a multisystemic autosomal dominant genetic disorder, inherited or sporadic, occurring in about 1/3 000 births.^13–15^ NF1 results from mutations in the *NF1* tumor suppressor gene, encoding neurofibromin protein, a negative regulator of RAS-mitogen-activated protein kinase (RAS-MAPK) pathways.^16^ Patients with NF1 manifest peripheral nerve sheath tumors such as cutaneous and plexiform neurofibromas (cNFs and pNFs), as well as hyperpigmentation on the skin or café-au-lait macules (CALMs), learning disabilities, and bone abnormalities.^17^ Half of NF1 patients exhibit bone-related symptoms, which can be generalized or focal, the latter being associated with significant morbidity and including long bone pseudarthrosis or spine deformity.^18^ Spine deformity including scoliosis is the most common type of bone manifestation in NF1 and mostly appears during childhood and early adolescence.^19^ Mild deformities are classified as non-dystrophic scoliosis, while the more severe form, dystrophic scoliosis, progresses rapidly and is associated with osseous changes, such as vertebral wedging, rotation, scalloping, fusion, and rib penciling.^20–23^ Other types of spine deformity reported in patients with NF1 are kyphosis and kyphoscoliosis.^12^ Treatments for NF1 spine deformity include physical therapy, bracing, or surgical interventions for severe and progressive cases.^12^ Due to a limited understanding of the pathogenesis, no pharmacological treatments have been tested in clinical trials or approved.

NF1 lesions, such as neurofibromas and CALMs, result from *NF1* biallelic inactivation in Schwann cells and melanocytes, respectively.^24–26^ Recently, Perrin et al. identified *NF1* biallelic inactivation in Schwann cells and skeletal stem progenitor cells (SSPCs) in congenital pseudarthrosis of the tibia.^27^ *NF1* biallelic inactivation has been reported in NF1 spine deformity, but the specific cell types affected have not been identified.^28^ Clinically, an increased incidence of dystrophic scoliosis has been reported adjacent to paraspinal plexiform neurofibromas (ppNFs).^29^ Yet, spine deformity in patients with NF1 can also develop in the absence of ppNFs. Overall, the cellular origin and underlying pathogenic mechanism of NF1 spine deformity remain unclear.

Several NF1 mouse models highlighted the potential role of *Nf1*-deficient bone cells in spine deformity.^22,30–32^ However, these models did not recapitulate the complexity of NF1 symptoms to investigate the etiopathology of NF1 spine phenotype. Among the NF1 mouse models described so far, the *Prss56-Nf1* KO mouse model is the only model reproducing various NF1-related symptoms. Analyses of the *Prss56-Nf1* KO mice revealed that boundary cap (BC) cells, originating from the neural crest and expressing *Prss56*, are the cells of origin of cNFs and pNFs.^33^ In addition, Prss56-expressing cells give rise to skeletal stem progenitor cells and Schwann cells in the periosteum of long bones and *Prss56-Nf1* KO mice exhibit fibrous nonunion reminiscent of congenital pseudarthrosis of the tibia.^27^ These data suggested a potential shared cellular origin for NF1 neurological, dermatological, and skeletal manifestations.^27,33–35^

As it recapitulates the diversity of NF1 symptoms, the *Prss56-Nf1* KO mouse model is also a promising pre-clinical model to test pharmacological approaches for NF1 neurofibromas and bone manifestations.^27,36^ Strategies to treat NF1 symptoms mainly target the hyperactivity of RAS-MAPK pathways. Clinical trials using MEK inhibitors were successful, leading to the Food and Drug Administration (FDA) approving selumetinib as a treatment for inoperable pNFs.^37–39^ While MAPK kinase (MEK) inhibitors showed limited and variable results in mice carrying *Nf1* gene inactivation in bone cells,^40,41^ combining selumetinib and the Src homology 2 containing protein tyrosine phosphatase 2 (SHP2) inhibitor SHP099 successfully prevented tibial fibrous nonunion in the *Prss56-Nf1* KO mouse model.^27^ In this study, we aimed to investigate the pathogenic mechanisms of spine deformity in the *Prss56-Nf1* KO mouse model and to evaluate a pharmacological approach for spine deformity prevention. Micro-computed tomography (CT) analyses revealed that *Prss56-Nf1* KO mice manifest NF1-related spine deformity and lineage tracing identified osteoblasts derived from the Prss56 lineage as the affected cell types. The increase of traced *Nf1*-deficient cells in the vertebrae correlated with the increase in bone volume and with spine deformity in *Prss56-Nf1* KO mice. We next successfully prevented spine deformity in *Prss56-Nf1* KO mice using the combination of selumetinib and RMC-4550, MEK/SHP2 inhibitors.

## Results

### *Prss56-Nf1* KO mice recapitulate NF1 spine deformity

**Fig. 1:**
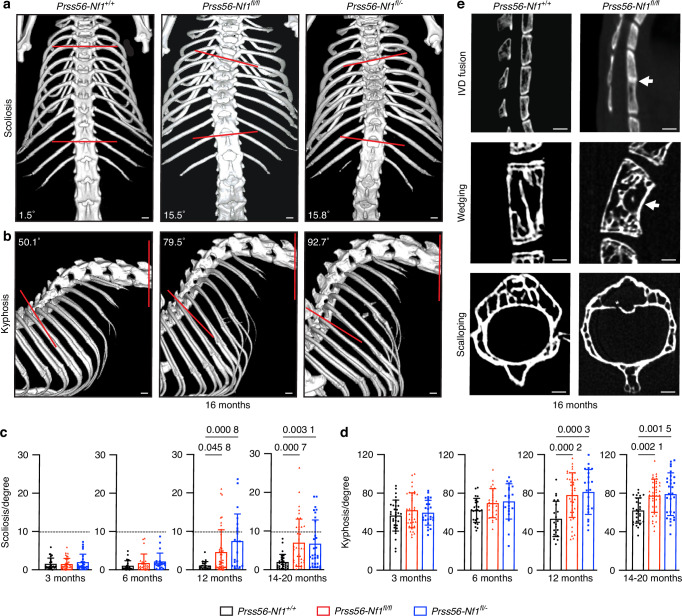
Spine deformity and vertebral anomalies in *Prss56-Nf1* KO mice. **a**, **b** In vivo micro-CT images of the spine in 16-month-old *Prss56*^*Cre*^*; R26*^*tdTom*^*; Nf1*^*+/+*^ (*Prss56-Nf1*^*+/+*^), *Prss56*^*Cre*^*; R26*^*tdTom*^*; Nf1*^*fl/fl*^ (*Prss56-Nf1*^*fl/fl*^) and *Prss56*^*Cre*^*; R26*^*tdTom*^*; Nf1*^*fl/-*^ (*Prss56-Nf1*^*fl/-*^) mice, illustrating scoliosis (a lateral deformity of 10 degrees or more) in coronal views (**a**) and kyphosis in sagittal views (**b**) in *Prss56-Nf1*^*fl/fl*^ and *Prss56-Nf1*^*fl/-*^ mutant mice. The respective Cobb angle measured between the two red lines drawn parallel to the endplate cartilage is indicated. **c**, **d** Quantitative analyses of the spine angle performed in 3-, 6-, 12- and 14-20-month-old mice showing the presence of scoliosis (**c**) and a significant increase of kyphosis angle (**d**) at 12 and 14-20 months of age in *Prss56-Nf1*^*fl/fl*^ and *Prss56-Nf1*^*fl/-*^ mutant mice compared to *Prss56-Nf1*^*+/+*^ control mice (*n* = 21-43 mice/group). Data are presented as mean ± SD. Statistical significance was determined using one-way ANOVA followed by Tukey’s multiple comparisons test. *P* < 0.05 was considered statistically significant. **e** Micro-CT images illustrating vertebral anomalies such as intervertebral disc (IVD) fusion (white arrow), wedging (white arrow), and scalloping (white asterisk) in *Prss56-Nf1*^*fl/fl*^ mutant compared to *Prss56-Nf1*^*+/+*^ control mice at 16 months of age. Scale bar, 1 mm

To determine whether *Prss56-Nf1* KO mice recapitulate NF1-associated spine deformity, we performed micro-CT analyses of the spinal column using the Cobb method in *Prss56*^*Cre*^*; R26*^*tdTom*^*; Nf1*^*fl/fl*^ (*Prss56-Nf1*^*fl/fl*^) and *Prss56*^*Cre*^*; R26*^*tdTom*^*; Nf1*^*fl/-*^ (*Prss56-Nf1*^*fl/-*^) mutant and *Prss56*^*Cre*^*; R26*^*tdTom*^*; Nf1*^*+/+*^ (*Prss56-Nf1*^*+/+*^) control mice from 3 to 20 months of age (Fig. 1a-d). In both *Prss56-Nf1*^*fl/fl*^ and *Prss56-Nf1*^*fl/-*^ mutant groups, mice carry *Nf1* biallelic inactivation in *Prss56*-expressing cells and their derivatives, while all other cells are wild type (*Prss56-Nf1*^*fl/fl*^ group) or *Nf1* heterozygote (*Prss56-Nf1*^*fl/-*^ group). We investigated the scoliosis phenotype through analyses performed in the coronal plane from the most cranial to the most caudal-tilted vertebrae within thoracic 2 (T2) to the lumbar 4 (L4) region. Scoliosis curvature, defined as a lateral curvature of 10° or more, was significantly increased in *Prss56-Nf1*^*fl/fl*^ and *Prss56-Nf1*^*fl/-*^ mutant mice from 12 months of age (Figs. 1a, c, S1a). Scoliosis was identified in 18.6% (8/43) and 30.77% (12/39) of *Prss56-Nf1*^*fl/fl*^ and 33.30% (7/21) and 27.8% (8/29) of *Prss56-Nf1*^*fl/-*^ mutant mice at 12 and 14-20 months of age, respectively (Fig. S1b). No scoliosis was detected in *Prss56-Nf1*^*+/+*^ control mice from 3 to 20 months of age (Figs. 1c, S1a, b). For kyphosis curvature, analyses were performed in the sagittal plane measuring the angle between the thoracic 8 (T8) to the lumbar 2 (L2) region. Results showed a significant increase in kyphosis spine angle in *Prss56-Nf1*^*fl/fl*^ and *Prss56-Nf1*^*fl/-*^ mutant compared to *Prss56-Nf1*^*+/+*^ control mice from 12 months of age (Fig. 1b-d). The kyphosis phenotype was not due to aging as it was progressive in *Prss56-Nf1*^*fl/fl*^ and *Prss56-Nf1*^*fl/-*^ mutant groups but not in *Prss56-Nf1*^*+/+*^ control mice (Fig. S1c, d). Interestingly, while scoliosis was detected in both male and female mice, the kyphosis phenotype was more severe in male compared to female mice as observed in NF1 patients^10^ (Fig. S2a, b). We also identified *Prss56-Nf1* mutant mice with scoliosis and high kyphosis angle (above 80°) recapitulating NF1 kyphoscoliosis spine deformity from 12 months of age (Fig. S2c). We identified 11.62% (5/43) and 12.82% (5/39) of *Prss56-Nf1*^*fl/fl*^, 14.28% (3/21), and 17.25% (5/29) of *Prss56-Nf1*^*fl/-*^ mutant mice manifesting kyphoscoliosis at 12 and 14-20 months of age, respectively (Fig. S2c). Moreover, as reported in patients with NF1, we detected vertebral anomalies in *Prss56-Nf1*^*fl/fl*^ and *Prss56-Nf1*^*fl/-*^ mutant mice, such as intervertebral disc fusion, vertebral wedging and scalloping, rib penciling, and sternum deformity at 14-20 months of age. Among the vertebral anomalies, intervertebral disc fusion was detected from 3 months of age (Figs. 1e, S2d-e, Table S1). Overall, these results show that *Prss56-Nf1*^*fl/fl*^ and *Prss56-Nf1*^*fl/-*^ mutant mice manifest spine deformity and vertebral anomalies reminiscent of spine deformity in patients with NF1.

### *Nf1* deficient osteoblasts are more abundant in the vertebrae of *Prss56-Nf1* KO mice

**Fig. 2:**
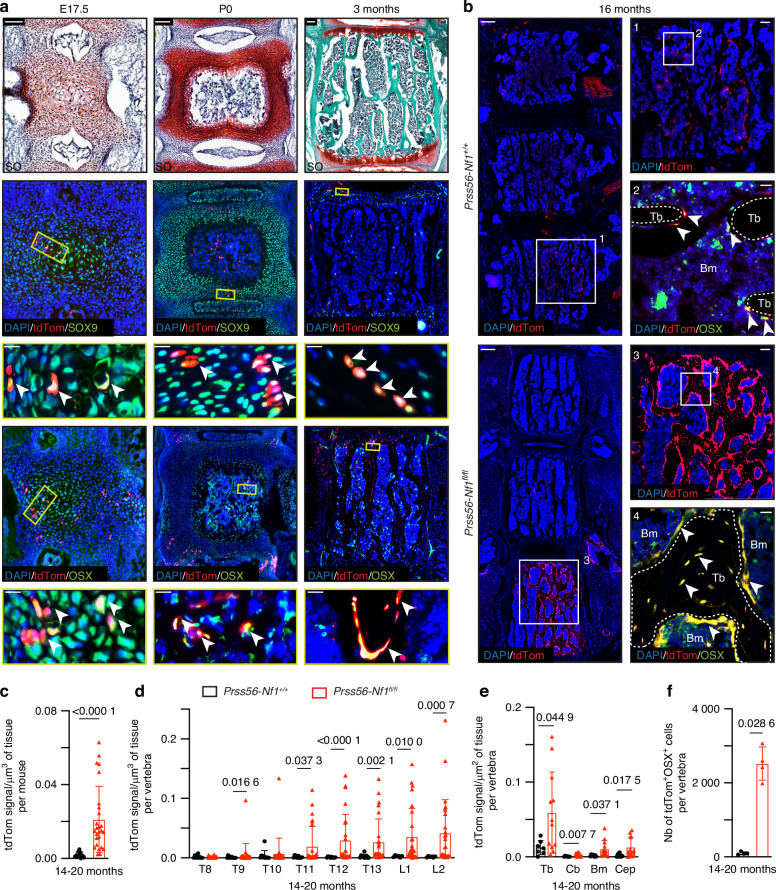
Increased *Nf1*^*−/−*^ osteoblasts/osteocytes in the vertebrae of *Prss56-Nf1* KO mice. **a** Vertebrae from *Prss56-Nf1*^*+/+*^ control mice stained with SafraninO (SO) and immunofluorescence showing tdTom^+^Sox9^+^ chondrocytes and tdTom^+^OSX^+^ hypertrophic chondrocytes (E17.5) and tdTom^+^OSX^+^ osteoblasts/osteocytes (P0, 3 months) (*n* = 3 samples). High magnification of yellow box area showing tdTom^+^ labeled cells (white arrowheads). Scale bar: low magnification 100 μm, high magnification 20 μm. **b** Left: Spinal column from *Prss56-Nf1*^*+/+*^ control and *Prss56-Nf1*^*fl/fl*^ mutant mice illustrating an irregular distribution and increased tdTom^+^ signal in mutant vertebrae. Right: High magnifications of the trabecular bone area showing more tdTom^+^ cells in mutant vertebrae (box 3) compared to control vertebrae (box 1) and more tdTom^+^OSX^+^ osteoblasts/osteocytes in mutant (box 4) compared to control (box 2) (white arrowheads). Scale bar: left boxes 200 μm; right boxes 1 and 3, 100 μm; and boxes 2 and 4, 25 μm. **c** Quantification showing significant increase of tdTom signal/volume of tissue per mouse in mutant mice (*n* = 10–26 mice/group). **d** Quantification of tdTom signal/volume of tissue in individual vertebrae from T8-L2 region showing a significant increase in the lower thoracic-lumbar region (T11-L2) of mutant mice (*n* = 10-26 vertebrae/group). **e** Quantification of tdTom signal/surface of tissue in trabecular bone (Tb), cortical bone (Cb), bone marrow (Bm) and cartilage endplate (Cep) in the vertebrae showing significant increase in Tb, Cb, Bm and Cep of mutant mice (*n* = 7-12 vertebrae/group). **f** Quantification of the number of tdTom^+^OSX^+^ cells in vertebrae showing a significant increase in mutant mice (*n* = 4 vertebrae/group). Data are presented as mean ± SD. Statistical significance was determined using Mann–Whitney U test. *P* < 0.05 was considered statistically significant

To investigate the role of Prss56-cell lineage in the NF1 spine phenotype, we traced tdTomato^+^ (tdTom^+^) *Prss56*-expressing boundary cap (BC) cells and their derivatives in the spine of *Prss56-Nf1*^*+/+*^ control and *Prss56-Nf1*^*fl/fl*^ mutant mice. We detected tdTom^+^ cells in the vertebrae from embryonic stage E12.5 until 14-20 months of age (Figs. 2 and S3). To determine if tdTom^+^ cells localized in the vertebrae were derived from BC expressing *Prss56*, we performed RNAscope in *Prss56-Nf1*^*+/+*^ control mice starting from embryonic stages E12.5 until 3 months of age. While tdTom^+^ BC cells expressed *Prss56* at E12.5, tdTom^+^ cells localized within vertebrae at E13.5, E14.5, and 3 months did not express *Prss56*, confirming that tdTom^+^ cells in the vertebrae are derived from early *Prss56*-expressing cells (Fig. S3a). Immunostaining showed that tdTom^+^ cells correspond to SOX9^+^ chondrocytes in E12.5 and E14.5 vertebrae and SOX9^+^ chondrocytes and OSX^+^ hypertrophic chondrocytes in E17.5 vertebrae (Figs. 2a, S3b). At P0 and 3 months, tdTom^+^ cells were mainly SOX9^+^ chondrocytes in endplate cartilage and OSX^+^ osteoblasts/osteocytes within the vertebral body (Fig. 2a). No tdTom^+^ cells were identified as TRAP^+^ osteoclasts at 3 months of age (Fig. S3c). Although we observed rare tdTom^+^ cells in the vertebrae of *Prss56-Nf1*^*+/+*^ mice, these tdTom^+^ cells were more abundant and irregularly distributed in the vertebrae of *Prss56-Nf1*^*fl/fl*^ mutant mice (Fig. 2b). Quantitative analyses confirmed a significant increase of the tdTom^+^ signal along the spinal column in *Prss56-Nf1*^*fl/fl*^ mutant compared to *Prss56-Nf1*^*+/+*^ control mice starting from embryonic stage E14.5 until 14-20 months of age (Figs. 2c, S3d). Analyses of individual vertebrae from thoracic T8 to lumbar L2 region revealed that tdTom^+^ signal is increased explicitly in the lower thoracic-lumbar region (T11-L2) and particularly in trabecular bone compared to cortical bone, bone marrow, cartilage end plate, intervertebral disc and periosteum (Figs. 2d, e, S3e). Further, analysis performed in individual vertebrae in the T11-L2 region showed a significant increase of tdTom^+^OSX^+^ osteoblasts/osteocytes in the vertebrae of 14-20-month-old *Prss56-Nf1*^*fl/fl*^ mutant mice (Fig. 2b, f). This increase in tdTom^+^ signal and tdTom^+^OSX^+^ osteoblasts/osteocytes was also observed in the *Prss56-Nf1*^*fl/-*^ mutant mice (Fig. S3f-i). These results show that *Prss56*-expressing cells give rise to bone cells in vertebrae and that *Nf1* biallelic inactivation in this lineage causes a significant increase of osteoblasts/osteocytes in the trabecular bone of *Prss56-Nf1* mutant vertebrae.

### *Nf1*-deficient osteoblasts exhibit osteogenic alterations and RAS-MAPK overactivation

**Fig. 3:**
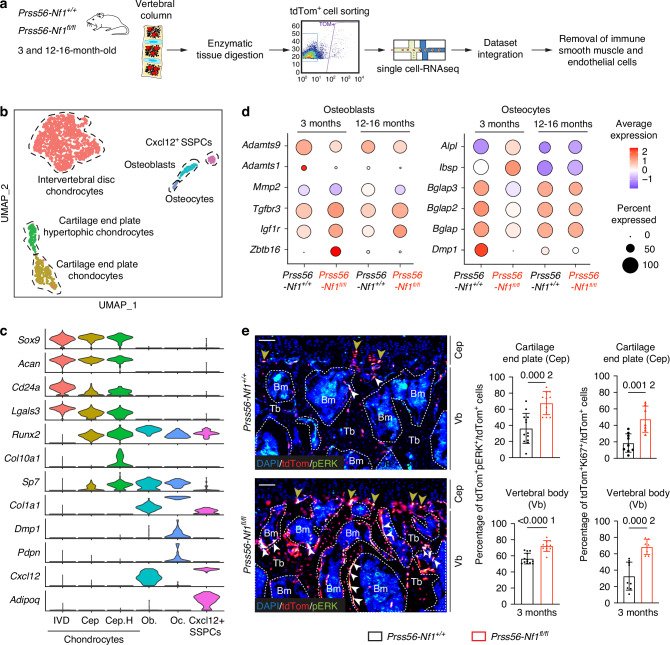
Single cell analyses of tdTom^+^ cells in the spine of *Prss56-Nf1* KO mice. **a** Experimental design for vertebral column processing and dataset integration. The vertebral column of *Prss56-Nf1*^*+/+*^ control and *Prss56-Nf1*^*fl/fl*^ mutant mice at 3 and 12-16 months of age were enzymatically digested and single tdTom^+^ cells were sorted and processed for single-cell RNA sequencing. Immune, smooth muscle and endothelial cells were identified and removed to focus the analysis on the cell populations of interest. **b** UMAP projection of color-coded clustering of the integration of control and mutant mice datasets. Six populations were identified and delimited by black dashed lines. **c** Violin plots of key marker genes of the different cell populations, IVD (intervertebral disc), Cep (cartilage endplate), Cep.H (Cartilage endplate hypertophic chondrocytes), Ob (osteoblasts), Oc (Osteoclasts), SSPCs (Skeletal stem progenitor cells). **d** Dot plot of the expression of genes related to osteogenesis in osteoblasts and osteocytes of control and mutant mice. Left: Expression of extracellular matrix remodeling genes (*Adamts9*, *Adamts1*, *Mmp2*) and osteogenic genes (*Tgfbr3*, *Igf1r*, *Zbtb16*) in osteoblasts. Right: Expression of early osteogenesis genes (*Alpl*, *Ibsp*) and late osteogenesis genes (*Bglap3*, *Bglap2*, *Bglap*, *Dmp1*) in osteocytes. **e** Left: Immunofluorescence images showing more tdTom^+^ cells co-expressing pERK in the cartilage endplate (yellow arrowheads) and vertebral body (white arrowheads) of mutant compared to control mice. Scale bar 100 μm. Right: Quantification of the percentage of tdTom^+^pERK^+^ and tdTom^+^Ki67^+^ cells revealing a significant increase in the cartilage endplate (Cep) and vertebral body (Vb) of mutant mice at 3 months of age. Tb = trabecular bone, Bm = Bone marrow (*n* = 7*-*13 vertebrae/group). Data are presented as mean ± SD. Statistical significance was determined using Mann–Whitney U test. *P* < 0.05 was considered statistically significant

To uncover the impact of *Nf1*^*−/−*^ mutation in osteoblasts of the Prss56 lineage, we performed single-cell RNA-sequencing (scRNA-seq) analyses on tdTom^+^ cells isolated from the vertebral columns of 3- and 12-16-month-old *Prss56-Nf1*^*+/+*^ control and *Prss56-Nf1*^*fl/fl*^ mutant mice (Fig. 3a). The integration of scRNA-seq datasets identified, among 1623 cells analyzed, nine clusters corresponding to nine cell populations: intervertebral disc chondrocytes expressing *Sox9* and *Cd24a*, cartilage endplate chondrocytes expressing *Sox9* and hypertrophic chondrocytes expressing *Sox9* and *Col10a1*, osteoblasts expressing *Runx2* and *Sp7*, osteocytes expressing *Dmp1*, skeletal stem progenitor cells (SSPCs) expressing *Cxcl12*, immune cells expressing *Ptprc*, smooth muscle cell (SMCs) expressing *Acta2* and endothelial cells (ECs) expressing *Cdh5* (Figs. 3b, c, S4a-c). Subsequent analyses were performed on the first six clusters after removing immune cells, SMCs, and ECs. Gene ontology (GO) analyses of upregulated genes in *Prss56-Nf1*^*fl/fl*^ mutant cells showed enrichment in gene sets related to connective tissue development, ossification, cartilage development, and osteoblast differentiation (Fig. S4d). The expression of the metalloproteinases *Adamts9*, *Adamts1*, and *Mmp2*, which are known to be involved in the regulation of bone formation, were decreased in *Prss56*-*Nf1*^*fl/fl*^ mutant compared to *Prss56*-*Nf1*^*+/+*^ control osteoblasts. In contrast, genes known to be involved in osteoblast differentiation *(Tgfbr3*, *Igf1r*, and *Zbtb16)* were upregulated in *Prss56-Nf1*^*fl/fl*^ mutant osteoblasts at 3 and 12-16 months of age (Fig. 3d). These osteogenic genes were also upregulated in *Prss56-Nf1*^*fl/fl*^ mutant cartilage endplate chondrocytes and hypertrophic cartilage endplate chondrocytes (Fig. S4e). In *Prss56-Nf1*^*fl/fl*^ mutant osteocytes, we observed an increased expression of early markers of osteogenesis (*Alpl*, *Ibsp*), while the expression of markers of late osteogenic maturation (*Bglap3, Bglap2, Bglap*, and *Dmp1*) were decreased compared to *Prss56-Nf1*^*+/+*^ control osteocytes (Fig. 3d). In parallel, histomorphometric analysis in undecalcified tissue showed a significant increase of osteoid surface in vertebrae of *Prss56-Nf1*^*fl/fl*^ mutant compared to *Prss56-Nf1*^*+/+*^ control mice, indicating a decrease in vertebral bone mineralization (Fig. S4f). We analyzed the proliferation and the activation of the MAPK pathway in *Nf1*-deficient cells using scores calculated from mouse gene sets in the gene ontology database. Proliferation and MAPK signaling activation were upregulated at 3 and 12-16 months of age in *Nf1*^*−/−*^ cartilage endplate chondrocytes and osteoblasts in *Prss56-Nf1*^*fl/fl*^ mutant compared to *Prss56-Nf1*^*+/+*^ control mice (Fig. S4g). Immunostainings on vertebrae of 3-month-old mice confirmed a higher percentage of tdTom^+^pERK^+^ and tdTom^+^Ki67^+^ cells in cartilage endplate and vertebral body of *Prss56-Nf1*^*fl/fl*^ mutant compared to *Prss56-Nf1*^*+/+*^ control mice, indicative of a higher RAS-MAPK pathway activity and proliferation (Fig. 3e). Collectively, these changes observed in *Nf1-*deficient tdTom^+^ osteoblasts in vertebrae indicate an acceleration in the early stages of osteogenesis followed by an impairment of the late stages of osteogenesis marked by a mineralization defect.

### Increased number of *Nf1*-deficient osteoblasts leads to changes in vertebral bone parameters and kyphosis spine curvature

**Fig. 4:**
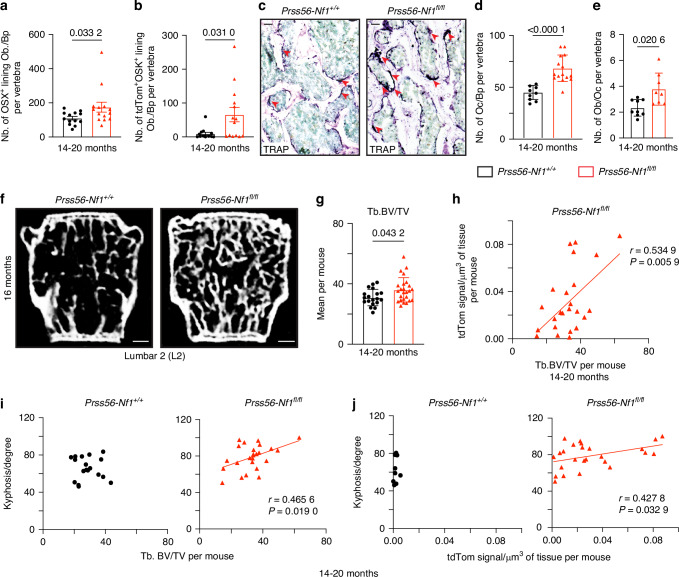
Increased number of *Nf1*^−/−^ osteoblasts correlates with changes in vertebral bone parameters and kyphosis spine curvature. **a** Quantification of the number of OSX^+^ lining osteoblasts per bone perimeter in vertebrae showing a significant increase in *Prss56-Nf1*^*fl/fl*^ mutant mice compared to *Prss56-Nf1*^*+/+*^ control at 14-20 months of age (*n* = 13-14 vertebrae/group). **b** Quantification of the number of tdTom^+^OSX^+^ lining osteoblasts per bone perimeter in vertebrae showing a significant increase in mutant mice (*n* = 12-14 vertebrae/group). **c** Vertebrae stained with TRAP illustrating more osteoclasts (dark purple) in mutant mice. Scale bar 50 μm **d** Quantification of the number of osteoclasts per bone perimeter in vertebrae showing a significant increase in mutant mice (*n* = 9-15 vertebrae/group). **e** Increased ratio of the number of lining osteoblasts/osteoclasts in mutant mice (*n* = 8-9 vertebrae/group) **f** Ex-vivo high-resolution micro-CT images of L2 vertebrae, illustrating increased trabecular bone (Tb) in mutant mice. Scale bar 0.1 mm. **g** Ex-vivo high-resolution analyses per mouse showing increased trabecular bone volume/total volume (Tb. BV/TV) in mutant mice (*n* = 19-25 mice/group). Data are presented as mean ± SD. Statistical significance was determined using Mann–Whitney U test. *P* < 0.05 was considered statistically significant. **h** Positive correlation between mean of trabecular bone volume/total volume (Tb. BV/TV) and tdTom signal/volume of tissue per mouse in mutant mice (*n* = 25 mice). **i** Correlation analyses performed between the mean of trabecular bone volume/total volume (Tb. BV/TV) per mouse and kyphosis spine curvature per mouse showing left: no significant correlation in control mice (*n* = 18 mice), right: positive correlation in mutant mice (*n* = 25 mice). **j** Correlation analyses performed between the mean of tdTom signal/volume of tissue per mouse and kyphosis spine curvature per mouse showing left: no significant correlation in control mice (*n* = 10 mice), right: positive correlation in mutant mice (*n* = 25 mice). Correlation was evaluated using Pearson’s correlation coefficient

Next, we sought to determine the consequences of *Nf1* loss in osteoblasts of the Prss56 lineage on vertebral bone parameters. In addition to the increase in total tdTom^+^OSX^+^ osteoblasts/osteocytes, we observed a significant increase in the number of OSX^+^ lining osteoblasts and tdTom^+^OSX^+^ lining osteoblasts in the vertebrae of *Prss56-Nf1*^*fl/fl*^ mutant compared to *Prss56-Nf1*^*+/+*^ control vertebrae at 14-20 months of age (Fig. 4a, b). Analyses of TRAP^+^ cells showed a significant increase in the number of osteoclasts per trabecular bone perimeter in the vertebrae of *Prss56-Nf1*^*fl/fl*^ mutant mice at 14-20 months of age (Fig. 4c, d). However, the ratio of lining osteoblasts/osteoclasts number per vertebra remained significantly higher in *Prss56-Nf1*^*fl/fl*^ mice, indicating increased bone formation in mutant vertebrae (Fig. 4e). High-resolution micro-CT scan measurements showed a significant increase in trabecular BV/TV of *Prss56-Nf1*^*fl/fl*^ mutant compared to *Prss56-Nf1*^*+/+*^ control mice at 3 and 14-20 months of age but no changes in cortical bone parameters (Figs. 4f, g, S5a-b). The increased trabecular BV/TV was correlated with the increased tdTom^+^ signal in the vertebrae of *Prss56-Nf1*^*fl/fl*^ mutant mice in the region T11-L2 (Fig. 4h). Due to the variability in the distribution of the tdTom^+^ signal along the vertebral column, we performed correlation analyses in individual vertebrae. In the vertebrae showing higher tdTom^+^ signal in the lower thoracic-lumbar region, such as L2, we observed a significant correlation between increased tdTom^+^ signal and increased trabecular bone parameters, including bone volume, number, and thickness. Conversely, no such correlation was detected in the upper thoracic vertebrae such as T8, where the tdTom^+^ signal was lower (Fig. S5c). Additionally, in *Prss56-Nf1*^*fl/fl*^ mutant mice, a significant correlation was found between increased tdTom^+^ signal and increased kyphosis angle, as well as between increased trabecular BV/TV and increased kyphosis angle. These correlations were absent in *Prss56-Nf1*^*+/+*^ control mice (Fig. 4i, j). These findings indicate that an increase in *Nf1*-deficient osteoblasts is associated with higher trabecular BV/TV in the vertebrae and kyphosis spine deformity in *Prss56-Nf1*^*fl/fl*^ mutant mice.

### The presence of paraspinal plexiform neurofibromas does not correlate with spine deformity in *Prss56-Nf1* KO mice

Given that *Prss56-Nf1* KO mice faithfully recapitulate paraspinal plexiform neurofibromas (ppNFs),^33^ which have been suspected to contribute to NF1 spine deformity,^42^ we further investigated the link between spine deformity and ppNFs. We analyzed spinal cord sections of *Prss56-Nf1*^*fl/fl*^ mutant mice to detect the paraspinal tumors identified as enlarged tdTom^+^ dorsal root ganglia compressing the spinal cord (Fig. S6a). *Prss56-Nf1*^*fl/fl*^ mutant mice were separated into two groups based on the presence or absence of ppNFs. We observed no significant differences in scoliosis and kyphosis spine deformities in *Prss56-Nf1*^*fl/fl*^ mutant mice with or without ppNFs (Fig. S6b). These results indicate that the development of paraspinal neurofibromas does not influence the prevalence of spine deformities in *Prss56-Nf1*^*fl/fl*^ mutant mice.

### Combined MEK and SHP2 inhibition prevents spine deformity in *Prss56-Nf1* KO mice

**Fig. 5:**
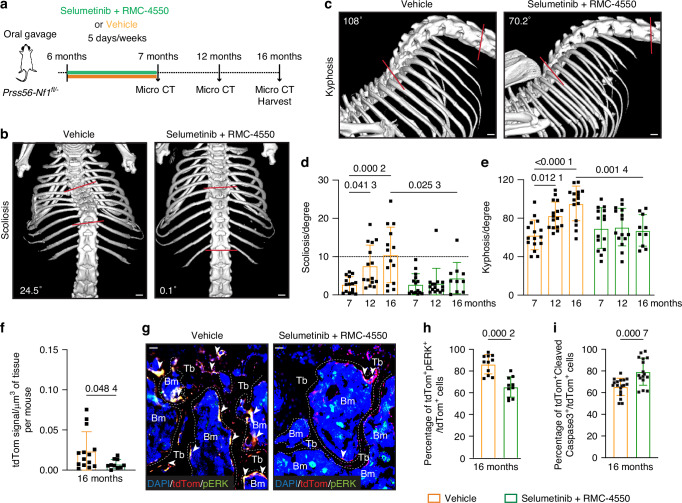
Combined MEK/SHP2 inhibition prevents spine deformity in *Prss56-Nf1* KO mice. **a** Experimental design. *Prss56-Nf1*^*fl/-*^ mice were treated by oral gavage with selumetinib (MEK inhibitor) and RMC-4550 (SHP2 inhibitor) or vehicle five days/week for four weeks starting at 6 months of age. Micro-CT scans were performed in vivo at 7-, 12-, and 16 months of age. In vivo micro-CT images of the spine in 16-month-old *Prss56-Nf1*^*fl/-*^ mutant mice treated with vehicle or selumetinib and RMC-4550 illustrating scoliosis in coronal view (**b**) and kyphosis in sagittal view (**c**) in mice treated with vehicle, and absence of scoliosis (**b**) and kyphosis (**c**) in selumetinib and RMC-4550 treated mice. The respective Cobb angle measured between the two red lines drawn parallel to the endplate cartilage of most tilted vertebrae is indicated. Scale bar: 1 mm. Quantitative analyses of the spine angle performed in 7-, 12-, and 16-month-old mutant mice showing (**d**) the presence of scoliosis in 33.3% (5/15) and 50% (7/14) of mice treated with vehicle and 6.6% (1/15) and 10% (1/10) of selumetinib and RMC-4550 treated mice at 12 and 16 months of age respectively and (**e**) a significant increase of kyphosis angle at 12 and 16 months of age in mice treated with the vehicle but not in mice treated with selumetinib and RMC-4550 (*n* = 10-15 mice/group). Data are presented as mean ± SD. Statistical significance was determined using two-way ANOVA followed by Tukey’s multiple comparisons test. *P* < 0.05 was considered statistically significant. **f** Quantification of tdTom signal/volume of tissue per mouse showing a significant decrease in selumetinib and RMC-4550 treated mutant mice (*n* = 10-14 mice/group). **g** Immunofluorescence images showing more tdTom^+^ cells co-expressing pERK (white arrowhead) in vehicle treated compared to selumetinib + RMC-4550 treated mutant mice at 16 months of age. Tb = trabecular bone, Bm = Bone marrow. Scale bar 50 μm. **h** Quantification of the percentage of tdTom^+^pERK^+^ cells revealing a significant decrease in the vertebrae of selumetinib + RMC-4550 treated mutant mice (*n* = 10-11/group). **i** Quantification of the percentage of tdTom^+^ Cleaved Caspase3^+^ cells revealing a significant increase in the vertebrae of selumetinib + RMC-4550 treated mutant mice (*n* = 16-18 vertebrae/group). Data are presented as mean ± SD. Statistical significance was determined using Mann–Whitney U test. *P* < 0.05 was considered statistically significant

To explore potential pharmacological approaches for NF1 spine deformity, we targeted the hyperactivated RAS-MAPK pathway in 6-month-old *Prss56-Nf1* KO mice at the onset of spine deformity. Given the limited success of MAPK kinase (MEK) inhibitors in NF1 bone phenotypes, Perrin et al. tested an approach using a MEK inhibitor combined with an inhibitor of Src homology 2 containing protein tyrosine phosphatase 2 (SHP2) to target the MAPK pathway upstream of RAS.^27^ This combined treatment significantly improved tibial bone union in *Prss56-Nf1* mutant mice compared to SHP2 or MEK inhibitor alone. Thus, we tested the efficacy of combining the MEK inhibitor selumetinib^39^ and the SHP2 inhibitor RMC-4550^43^ in NF1 spine deformity. We treated *Prss56-Nf1*^*fl/-*^ mice, which reproduce the human genetic landscape of NF1, with combined selumetinib and RMC-4550 or vehicle by gavage for four weeks between 6 and 7 months of age (Fig. 5a). Combined treatment with selumetinib and RMC-4550 effectively prevented scoliosis in *Prss56-Nf1*^*fl/-*^ mice while 33.3% (5/15) and 50% (7/14) of vehicle-treated mice developed scoliosis by 12 and 16 months, respectively (Figs. 5b-d, S7a). One mouse with a scoliosis angle of 9.2° at 7 months presented scoliosis in the treated group. In addition, mice treated with combined selumetinib and RMC-4550 did not show progressive kyphosis angle at 12 and 16 months of age, whereas we found a significant increase in kyphosis angle in *Prss56-Nf1*^*fl/-*^ mice treated with the vehicle at 12 and 16 months of age (Figs. 5c-e, S7b). We quantified the tdTom^+^ signal in the vertebrae of *Prss56-Nf1*^*fl/-*^ mutant mice treated with vehicle or selumetinib and RMC-4550. Analyses of individual vertebrae from thoracic T8 to lumbar L2 region revealed that tdTom^+^ signal is significantly reduced in the lower thoracic-lumbar region (T11-L2) in *Prss56-Nf1*^*fl/-*^ mutant mice treated with selumetinib and RMC-4550 compared to mutant mice treated with vehicle (Figs. 5f, S7c). Immunostainings on vertebrae of 16-month-old mice showed a significant reduction in the percentage of tdTom^+^pERK^+^ cells and a significant increase in the percentage of tdTom^+^Cleaved Caspase3^+^ cells while no significant differences were detected in the percentage of tdTom^+^Ki67^+^ cells in *Prss56-Nf1*^*fl/-*^ mutant mice treated with selumetinib and RMC-4550 compared to vehicle-treated mice (Figs. 5g-i, S7d). Analyses showed no significant changes in the number of osteoclasts in *Prss56-Nf1*^*fl/-*^ mutant mice treated with combined inhibitor compared to vehicle-treated mice (Fig. S7e). Overall, these results show the efficacy of MEK-SHP2 inhibition in preventing the progression of spine deformities in *Prss56-Nf1* mutant mice, demonstrating its therapeutic potential.

## Discussion

In this study, we report that the *Prss56-Nf1* KO mouse model accurately recapitulates NF1-related spine deformity. Analyses performed on *Prss56-Nf1* mutant mice revealed progressive scoliosis, kyphosis, and kyphoscoliosis, closely mirroring the spine deformities observed in patients with NF1. In *Prss56-Nf1* mutant mice, we also detected vertebral anomalies resembling structural anomalies observed in NF1. Interestingly, vertebral fusion appeared as early as 3 months of age, before detectable spine deformity, highlighting a critical period of vertebral development vulnerable to *Nf1* inactivation. Unlike previous models,^22,31^ where spine deformities were congenital and lacked the context of NF1 pathology, the *Prss56-Nf1* model captures both the postnatal onset of NF1-related spine deformity and the multisystemic complexity, making it a valuable tool for studying this condition in parallel with other NF1 symptoms. Despite the presence of ppNFs in the *Prss56-Nf1* model, we found no significant correlation between ppNFs and spine deformity. This suggests that cell alterations driven by the *Nf1* mutation, rather than mechanical compression or paracrine factors from tumors, play a critical role in spine deformities, shifting the focus toward targeting molecular pathways in affected cell types for treating NF1 spine deformity.

Lineage tracing revealed that tdTom^+^ cells from the Prss56 lineage contribute to vertebral bone formation, corresponding mainly to bone cells. This challenges the traditional view that neural crest-derived cells only contribute to the cranial bone. Another study further supports the broader potential of neural crest-derived populations to partially contribute to trunk skeletal formation.^44^ Interestingly, we observed variability in the distribution of tdTom^+^ cells across different vertebrae, which may be influenced by regional factors that regulate their fate during development. To build on this observation, further investigation is needed to explore the mechanisms driving and influencing the behavior of *Prss56*-expressing cells and their derivatives. This finding opens new avenues for understanding the role of neural crest-derived subpopulations and, specifically, BC derivatives in normal and pathological conditions and will be relevant for explaining the heterogeneity of clinical manifestations observed in NF1 patients.

The increase in tdTom^+^ signal in mutant vertebrae from embryonic stages to adulthood, and particularly the increase of tdTom^+^ osteoblasts within the trabecular bone, suggests that early *Nf1* mutations in Prss56 lineage influence proliferation, likely due to disrupted RAS-MAPK signaling. The increased activity in osteoblasts proliferation and RAS-MAPK signaling was confirmed by single-cell RNA-seq analysis. In the *Prss56-Nf1* model, the *Nf1*^*−/−*^ osteoblasts maintained a high RAS-MAPK pathway activity throughout adulthood, and this was associated with increased RAS-MAPK pathway activity in mutant cartilage endplate chondrocytes and progenitor populations. *Nf1* mutation in osteoblasts also affected their osteogenic differentiation with a delay in maturation and mineralization, suggesting alterations in mutant vertebrae ossification.

Both the alteration in osteoblast cell function and increased irregular distribution of tdTom^+^ osteoblasts across the mutant vertebrae likely contribute to the variability in NF1 spine phenotypes in mutant mice. This led us to analyze individual vertebrae and establish a correlation between increased tdTom^+^ signal in trabecular bone and increased trabecular bone volume, while cortical bone remained unaffected. The increase in the number of tdTom^+^ osteoblasts in vertebrae paralleled the increase in the number of osteoclasts. However, their ratio remained significantly higher in mutant mice, indicating an imbalance between bone formation and resorption, and leading to excessive trabecular bone mass. Increased bone mass aligns with results in the *Col1a1Cre* model, where *Nf1* is inactivated in osteoblasts.^30^ In contrast, the *Col2a1Cre* model, with *Nf1* mutation in chondrocytes and osteoprogenitors, showed reduced bone mass.^31^ We revealed a significant correlation between the increase in the tdTom^+^ signal or the increase in trabecular bone volume with the kyphosis angle in *Prss56-Nf1* mutant mice, suggesting a link between the number of *Nf1*-deficient cells in vertebrae and the severity of the phenotype. Overall, the cascade of changes from the cellular level to the tissue structure elucidates a critical pathway through which *Nf1* mutations lead to spinal deformity involving *Nf1*^*−/−*^ osteoblasts of the Prss56 lineage as the cellular origin. Further investigation is essential to explore the cell origins of NF1-related spine deformity in patients and to bridge the gap between findings in *Prss56-Nf1* KO mouse models and in patients with NF1, widening the way for therapeutic strategies.

The *Prss56-Nf1* model effectively replicates both neural and skeletal symptoms, including not only plexiform and cutaneous neurofibromas, skin hyperpigmentation, and tibial pseudarthrosis,^27,33^ but also spine deformity. This reflects the complexity of NF1 disease and points to a critical role of BC cells as a common cellular origin for the diverse NF1 symptoms. Yet, different pathogenic mechanisms appear to drive these symptoms. Tibial pseudarthrosis involves both the paracrine effects of *Nf1*^*−/−*^ Schwann cells and cell-autonomous effect on *Nf1*^*−/−*^ skeletal stem progenitors. We found that spine deformity in the *Prss56-Nf1* mouse model was mainly associated with the presence of *Nf1*^*−/−*^ osteoblasts. These findings emphasize the complexity and variability of pathogenic mechanisms in the *Prss56-Nf1* model of NF1-associated bone disease, suggesting that different skeletal regions may be vulnerable to distinct pathogenic processes, even within the same individual. This highlights the importance of addressing both neural and skeletal components when developing treatments for NF1 and, most importantly, the consequences of *Nf1* loss in diverse cell types and physiological processes. This prompted us to explore a pharmacological approach targeting this pathway postnatally to reduce the progression of the symptoms efficiently. We treated *Prss56-Nf1*^*fl/-*^ mutant mice with a combination of selumetinib and RMC-4550 to block hyperactivated RAS pathways. This combination effectively prevented the progression of spine deformities and reduced the tdTom^+^ signal in vertebrae, further indicating that tdTom^+^ cells play a significant role in the manifestation of spine deformity. Likely, the reduction of tdTom^+^ signal in treated mutant mice may have contributed to maintaining vertebral bone volume equilibrium along the vertebral column. These results suggest that targeting the RAS-MAPK pathway could be a promising strategy to alleviate NF1 spine deformity in clinical settings.

## Methods

### Mice

C57BL/6, *R26tdTomato* (*R26*^*tdTom*^),^45^ *Nf1*^*flox*^ (*Nf1*^*fl*^), *Nf1*-knock out (*Nf1*^*-*^)^46^ were obtained from Jackson Laboratory (Bar Harbor, ME). *Prss56*^*Cre*^ mice were provided by Piotr Topilko.^33,34^ All mice were housed in a pathogen-controlled, ventilated cage with 12:12-h light: dark cycles and ad libitum access to water and food at the animal facilities at IMRB, Creteil. Genotypes were verified by PCR, utilizing tail DNA. Males and females were included in experimental groups with no specific randomization method. Controlled breeding was conducted to collect embryos at days E12.5, 13.5, 14.5, 17.5, and at birth (P0). Samples were labeled at the collection for blinded analyses.

### Micro-computed tomography (micro-CT)

Mice were anesthetized with a concentration of 2.5% isoflurane delivered in oxygen and scanned in vivo at 3, 6, 12, and 14–20 months of age using a Quantum FXCaliper micro-CT system (PerkinElmer, Waltham, MA) at 90 kV, 160 μA. For most mice, follow-up scans were performed at multiple time points to monitor changes over time. The last scan was performed at 16-17 months of age in *Prss56-Nf1*^*+/+*^ control mice. In *Prss56-Nf1*^*fl/fl*^ and *Prss56-Nf1*^*fl/-*^ mutant mice, the last scan was performed from 14 months of age, depending on the prevalence of plexiform neurofibromas progressing to malignant peripheral nerve sheath tumors that required earlier sacrifice of the animals. A 40 mm field of view (FOV40) and 80 μm voxel size were used to evaluate the spinal column. Spine angles were quantified in 2D projection using Horos software and the Cobb technique.^47^ Parallel lines were drawn from the superior to the inferior-most tilted vertebral endplate adjusted at the level of scoliosis curvature on a coronal view between the thoracic (T2) to the lumbar (L4) region. Cobb angles greater than or equal to 10 degrees were considered scoliosis. Kyphosis angle was measured on sagittal view between lines drawn parallel to the superior thoracic (T8) and the inferior lumbar (L2) vertebral endplates. For high-resolution ex vivo scans, vertebrae were collected postmortem, scanned at 10 μm voxel size (FOV 5 mm), and analyzed with CTan (v1.17.7.2, Bruker, Germany). Cortical and trabecular compartments were segmented manually on 1 mm sections, and bone parameters including bone volume/ total volume (BV/TV, %), trabecular number (Tb.N, mm^−1^) and trabecular thickness (Tb.Th, μm) were analyzed in individual vertebrae in the region from thoracic (T8) to lumbar (L2).

### Tissue sample processing and histology

Samples were collected at embryonic day 12.5, 13.5, 14.5, 17.5, neonatal (P0), and adult (3 and 14–20 months) stages. The spinal column was fixed in 4% paraformaldehyde PFA (sc-281692, CliniSciences) for 4 or 24 h with constant shaking at 4 °C, rinsed in PBS, and decalcified in 19% EDTA (EU00084, Euromedex) at pH = 7.4 for 1 day (P0) or 10 days (adult), while other samples remained undecalcified as required from the experimental design. Samples were cryoprotected in 30% sucrose (200-301-B, Euromedex) for 24 h, embedded in cryoprotectant freezing tissue medium OCT (F/TFM-C, MM France), sectioned longitudinally (T3–T13, L1–L6), and stored at –20 °C. Decalcified sections were cut at 7 μm (embryo) or 10 μm (P0/adult). Undecalcified sections were prepared at 5 μm using Kawamoto’s Film Method Cryofilm type 3 C (16UF).^48^ Sections were stained with Safranin’O for cartilage, Masson’s Trichrome for bone, TRAP for osteoclasts, and Von Kossa/Van Gieson for mineralization. Every thirtieth of sections was mounted with Fluoromount-G DAPI staining to trace tdTom^+^ cells in the vertebrae and to detect the ppNFs. Images were acquired with ZEISS Axioscan 7 Colibri and Zeiss Imager D1 AX10 light microscope (Carl Zeiss Microscopy GmbH).

### Histological staining

#### Masson’s trichrome (TC)

Sections were hydrated, stained in hematoxylin, Red Mallory, phosphomolybdic acid, light green and finally dehydrated before mounting as described.^27^

#### Safranin’O (SO)

Sections were hydrated, stained with Weigert’s solution, Fast Green, Safranin’O and then dehydrated and mounted as described.^27^

#### Tartrate-resistant acid phosphatase (TRAP)

Sections were hydrated for 5 min, placed in pre-warmed TRAP staining solution mix (387 A, Sigma Aldrich), and incubated at 37 °C for 20 min. After counterstaining with 0.01% Methylene blue for 1 min, rinsing in distilled water, sections were mounted with Fluoromount-G mounting medium with DAPI (00-4959-52, Life Technologies). Purple-labeled cells along the trabecular bone surface were counted in 3 individual vertebrae/section/mouse using QuPath. Osteoclasts number was normalized to the bone perimeter (mm) per vertebra.

#### Von Kossa/Van Gieson

Undecalcified sections were rehydrated in distilled water for 5 min and incubated with 5% silver nitrate solution light protected for 20 min. Slides were rinsed in distilled water, treated with sodium carbonate+formaldehyde for 2 min, and washed. Counterstaining was performed with Van Gieson’s stain (mixture of saturated picric acid and acid fuchsin) for 10 min, followed by washes, dehydration, and coverslipping. Calcium deposits appeared black, collagen red/pink, and yellow-brownish muscle/bone marrow. Counting was performed in 3–5 vertebrae/section/mouse using QuPath. The osteoid surface was normalized to the total bone surface (mm^2^) per vertebra.

### Immunostaining

Cryosections were defrosted at room temperature for 30 min, light protected, rehydrated in PBS for 5 min. Sections were incubated for 1 h at room temperature in 5% serum/0.25% Triton PBS with primary antibodies Sox9 (ab185230, Abcam) dilution 1:1 000, OSX (ab22552, Abcam) dilution 1:200, pERK (9101S, Ozyme) dilution 1:200, Cleaved Caspase-3 (Asp175, Ozyme) dilution 1:200, or Ki67 (ab15580, Abcam) dilution 1:200, overnight at 4 °C. Antigen retrieval (citrate buffer, 95 °C–20 min, 4 °C–20 min) was applied for OSX and pERK. Secondary antibody Alexa Fluor 488 goat anti-rabbit Ig (A-11034, Invitrogen) dilution 1:1 000 was applied at room temperature for 1 h. Sections were mounted with Fluoromount-G/DAPI (00-4959-52, Life Technologies). Positive controls validated stainings.

### Fluorescent signal quantification

The volume of tdTom^+^ signal in the vertebral body (including trabecular, cortical bone, and cartilage endplate) was measured on 3 sections 300 μm apart as described,^27^ captured using 10X objective, and the tdTom^+^ surface was measured using Zen Software v1.1.2.0 (Carl Zeiss Microscopy GmbH). Volumes of tdTom^+^ signal per vertebra and total tissue were calculated using the formula: ${volume}=\,\frac{1}{3}h{\sum }_{1}^{n-1}({A}_{i}+{A}_{i+1}+\sqrt{{A}_{i}* {A}_{i+1}})$ with A_i_ and A_i+1_: with A_i_ and A_i+1_ being the areas of the vertebral body, h distance between A_i_ and A_i + 1_ (300 μm) and n the total number of sections analyzed.

The tdTom^+^ signal surface were quantified using Zen Software v1.1.2.0 in the trabecular and cortical bone, intervertebral disc, cartilage endplate, and periosteum on vertebrae captured with 20X objective, and normalized on the total tissue surface.

OSX^+^ and tdTom^+^OSX^+^ lining osteoblasts along the trabecular bone were counted and normalized to the trabecular bone perimeter (mm) using QuPath. The positive cell number in vertebrae was determined for each section.

The number of tdTom^+^pERK^+^ , tdTom^+^Cleaved Caspase3^+^ , tdTom^+^Ki67^+^ cells in the cartilage endplate and vertebral body were counted separately in each vertebra and normalized to the tdTom^+^ cell number using QuPath. The cell percentage was determined for each vertebra.

### RNAscope in situ hybridation

RNAscope® Multiplex Fluorescent Assay V2 (Biotechne) was used to detect *Prss56* and *tdTom* transcripts at E12.5-14.5 and 3 months as described.^27^ Briefly, samples were fixed in 4% PFA, cryoprotected, sectioned at 10 μm, and processed following manufacturer’s guidelines: 15 min post-fixation in 4% PFA, ethanol dehydration, 10 min of H_2_O_2_, 5 min of target retrieval at 95 °C, and 30 min of protease III treatment. After hybridization and revelation, the sections were mounted under a glass coverslip with Prolong Gold Antifade (P10144, Thermo Fischer). For adult sections, target retrieval and protease III treatment were replaced by incubation in ACD custom reagent for 30 min at 40 °C. Sections were pictured and analyzed using Zeiss LSM800.

### Single-cell RNA sequencing tissue processing

Thoracic-lumbar vertebrae (T11-L2) with detectable tdTom^+^ signal were collected at 3- and 12-16-month-old from *Prss56-Nf1*^*+/+*^ control and *Prss56-Nf1*^*fl/fl*^ mutant mice. Muscle, spinal cord, and dorsal root ganglia were removed and confirmed by observation under a fluorescent microscope. The tissues were minced using a scalpel and scissors, and digested with 1 mg/mL of Collagenase (C6885, Merck) and 2 mg/mL of Dispase (D4693, Merck) in PBS for 20 min at 37°. Later, 10 μL/mL of 1% DNAse (WOLS02007, Serlabo, France) were added and incubated for 10 min at 37 °C. The supernatant was filtered and collected in sorting buffer composed of MEM-alpha and 10% FBS. The remaining tissue underwent a second step of digestion with 2 μL/mL of 1% DNAse for 20 min at 37 °C. Collected cells were centrifuged at 4 °C for 10 min, supernatant was discarded, and the cells were resuspended in sorting buffer and incubated for 30 min with antibodies for immune and endothelial cells CD11b-BV786 (BD Biosciences 740861), CD31- BV786 (BD Biosciences 740879), CD45-BV786 (BD Biosciences 564225) on ice and light protected. The cells were washed, resuspended in sorting buffer, and Sytox Blue (S34857, Thermo Fisher Scientific) was added to stain dead cells. Using Influx Cell Sorter, the immune, endothelial, and dead cells were sorted and discarded, while tdTom^+^ cells were collected. A total of 4 000 to 20 000 cells were loaded into the 10x Chromium Controller and libraries were prepared with Chromium Single Cell Next GEM 3′ Library & Gel Bead Kit v3.1 (10x Genomics) according to the manufacturer’s instructions. Sequencing was performed using Nextseq550 (Illumina) followed by reads alignment on the mm10 mouse genome following the Cell Ranger count pipeline.

### Single-cell RNA sequencing analyses

Single-cell RNAseq analyses were performed using Seurat 5.0.3 and Rstudio v.2023.12.1 + 402.

### Filtering and clustering using Seurat

Aligned scRNAseq datasets were corrected for ambient RNA using SoupX v1.6.2 and the genes expressed in less than 5 cells were removed. Each dataset was filtered to retain only cells with more than 750 RNA counts, expressing more than 500 genes and expressing less than 7% of mitochondrial genes. We obtained approximately 607, 125, 472 and 418 cells from 3- and 12-16-month-old *Prss56-Nf1*^*+/+*^ control and *Prss56-Nf1*^*fl/fl*^ mutant mice, respectively. Integration of the datasets was performed using the CCA method. Contamination from immune cells, smooth muscle cell and endothelial cells were removed from the analyses. Clustering was performed using the first 20 principal components and a resolution of 0.4.

### Gene ontology (GO)

Differentially expressed genes in mutant vs control were obtained with Seurat’s FindMarkers function. Genes upregulated in the mutant with adj. *P* < 0.05 and log_2_FC > 0.5 were selected to do a gene ontology analysis using clusterProfiler (v4.10.1).

### MAPK pathway activation and proliferation score

Scores were calculated based on the average expression of the list of genes from the 2024 version of the Gene Ontology terms “Positive regulation of MAPK cascade” and “Positive regulation of cell population proliferation” using the AddModuleScore function.

### Selumetinib and RMC-4550 in vivo treatment

At 6 months of age, *Prss56-Nf1*^*fl/-*^ mutant mice were treated by daily oral gavage (5 days/week, 4 weeks). Mice received a combination of 25 mg/kg selumetinib (HY-50706, Clinisciences) and 30 mg/kg RMC-4550 (Revolution Medicines, Inc., Redwood City, CA), or vehicle. Selumetinib was dissolved in 0.5% methylcellulose/0.2% Tween-80 and RMC-4550 in a solution of 0.9% NaCl/2.13% 1 mol/L HCl. The vehicle group was treated with 0.9% NaCl/2.13% 1 mol/L HCl. Mice were scanned in vivo at 7, 12, and 16 months of age in the small Animal Platform of Paris Cité University (EA2496, Paris Cité), as described above.

### Statistical analyses

Data are reported as mean ± standard deviation with *n* representing mice or vertebrae. Statistical analyses were performed using GraphPad Prism. Statistical comparisons used for two groups were Mann-Whitney test, for ≥3 groups were one-way/two-way ANOVA with Tukey’s multiple comparisons post-hoc test or correlation/regression analysis. Correlation analysis and simple linear regression were performed to assess the correlation between parameters. *P* < 0.05 was considered significant. Tests are specified in figure legends. All experiments were replicated at least twice.

### Study approval

All procedures performed were approved by the Paris Est Creteil University Ethical Committee (APAFIS #50387-202312221643271 v8, #33818-2021110818301267 v5 and #27181-202009141201846 v5).

## Supplementary information

Supplemental material unmarked

## Data Availability

The single-cell RNA-seq datasets generated for this study are deposited in GEO (GSE305286).
