# Supplementary material for: Pharmacological inhibition of RAS pathway alleviates spine deformity in a mouse model of neurofibromatosis type 1
Source: Bone Res. 2025 Dec 16;13:103. doi: 10.1038/s41413-025-00492-3 (PMC12706011; doi:10.1038/s41413-025-00492-3)
Supplement: Supplementary file 1 — Supplemental material unmarked [file 41413_2025_492_MOESM1_ESM.pdf]

Pharmacological inhibition of RAS pathway alleviates spine deformity in a mouse model of Neurofibromatosis Type 1

Supplemental material

Supplemental Figures

Supplemental Figure 1: ***Prss56-Nf1*** KO mice recapitulate NF1 spine deformity

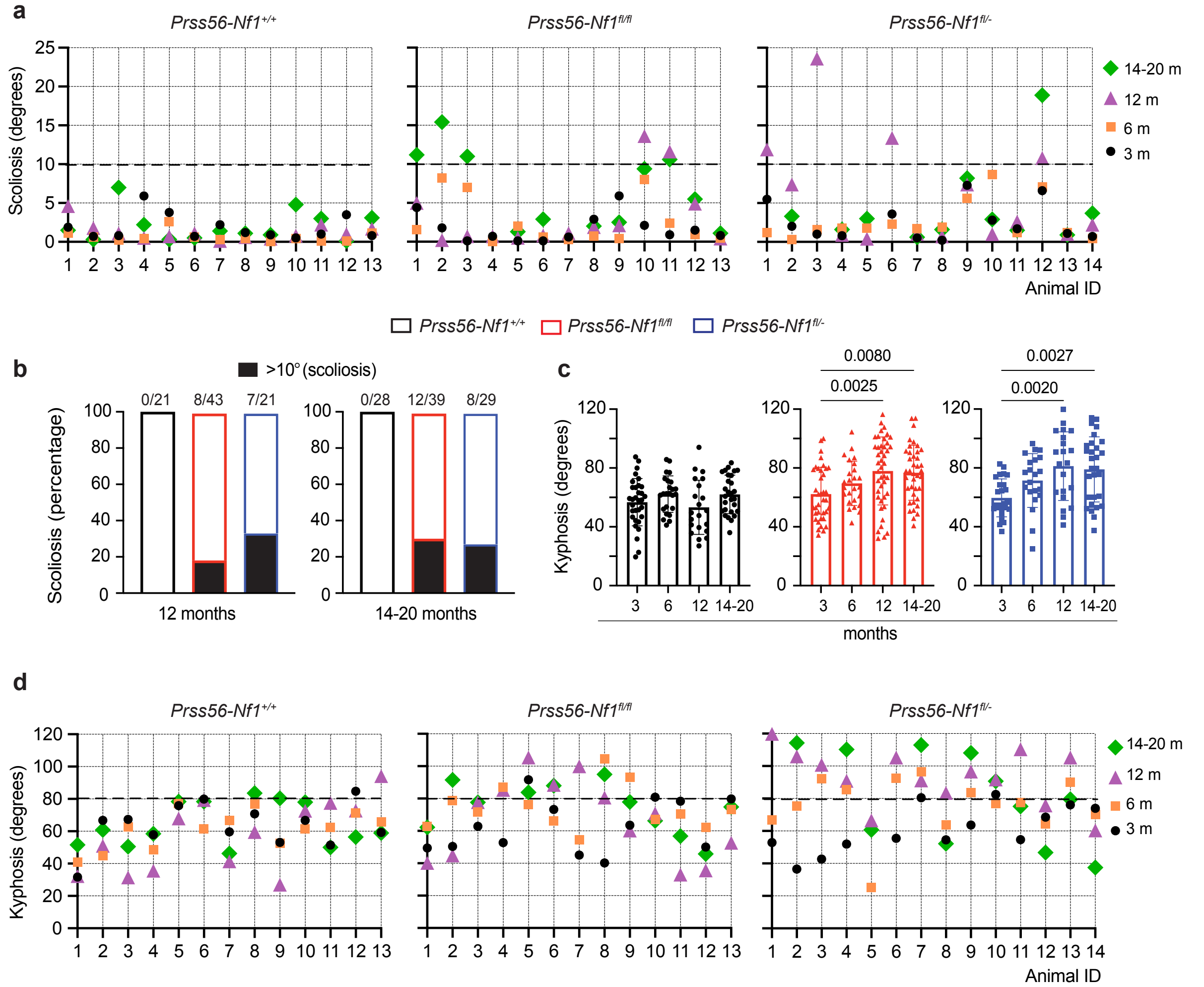

a. Graphs showing scoliosis angle followed overtime in individual mice from each genotype *Prss56-Nf1*$^{+/+}$ control and *Prss56-Nf1*$^{fl/fl}$ and *Prss56-Nf1*$^{fl/-}$ mutant mice. Scoliosis was defined as a lateral curvature >10°. Each animal ID present one mouse evaluated at 3-, 6-, 12- and 14-20 months of age. Scoliosis is present in *Prss56-Nf1*$^{fl/fl}$ and *Prss56-Nf1*$^{fl/-}$ mutant mice beginning at 12 months of age while *Prss56-Nf1*$^{+/+}$ control mice do not show scoliosis (n=13-14 mice/group). **b.** Graphs of percentage showing 0% (n= 0/21) and (n=0/28) of *Prss56-Nf1*$^{+/+}$ control mice, 18.6% (n=8/43) and 30.7% (n=12/39) of *Prss56-Nf1*$^{fl/fl}$ and 33.3% (n=7/21) and 27.5% (n=8/29) of *Prss56-Nf1*$^{fl/-}$ mutant mice with scoliosis at 12 and 14-20 months of age respectively. **c.** Analyses showing a progressive increase of kyphosis angle at 3, 6, 12, and 14-20 months of age in *Prss56-Nf1*$^{fl/fl}$ and *Prss56-Nf1*$^{fl/-}$ mutant, but not in *Prss56-Nf1*$^{+/+}$ control mice (n=21-43 mice/group). Data are presented as mean ± SD. Statistical significance was determined using one-way ANOVA followed by Tukey's multiple comparisons test. $p < 0.05$ was considered statistically significant. **d.** Graphs showing kyphosis angle followed overtime in individual mice from each genotype *Prss56-Nf1*$^{+/+}$ control and *Prss56-Nf1*$^{fl/fl}$ and *Prss56-Nf1*$^{fl/-}$ mutant mice. Each animal ID present one mouse evaluated at 3-, 6-, 12- and 14-20 months of age. Kyphosis angle >80° are present in *Prss56-Nf1*$^{fl/fl}$ and *Prss56-Nf1*$^{fl/-}$ mutant mice beginning at 12 months of age while *Prss56-Nf1*$^{+/+}$ control mice do not show kyphosis (n=13-14 mice/group).

Supplemental Figure 2: *Prss56-Nf1* KO mice recapitulate NF1 spine deformity associated with vertebral anomalies

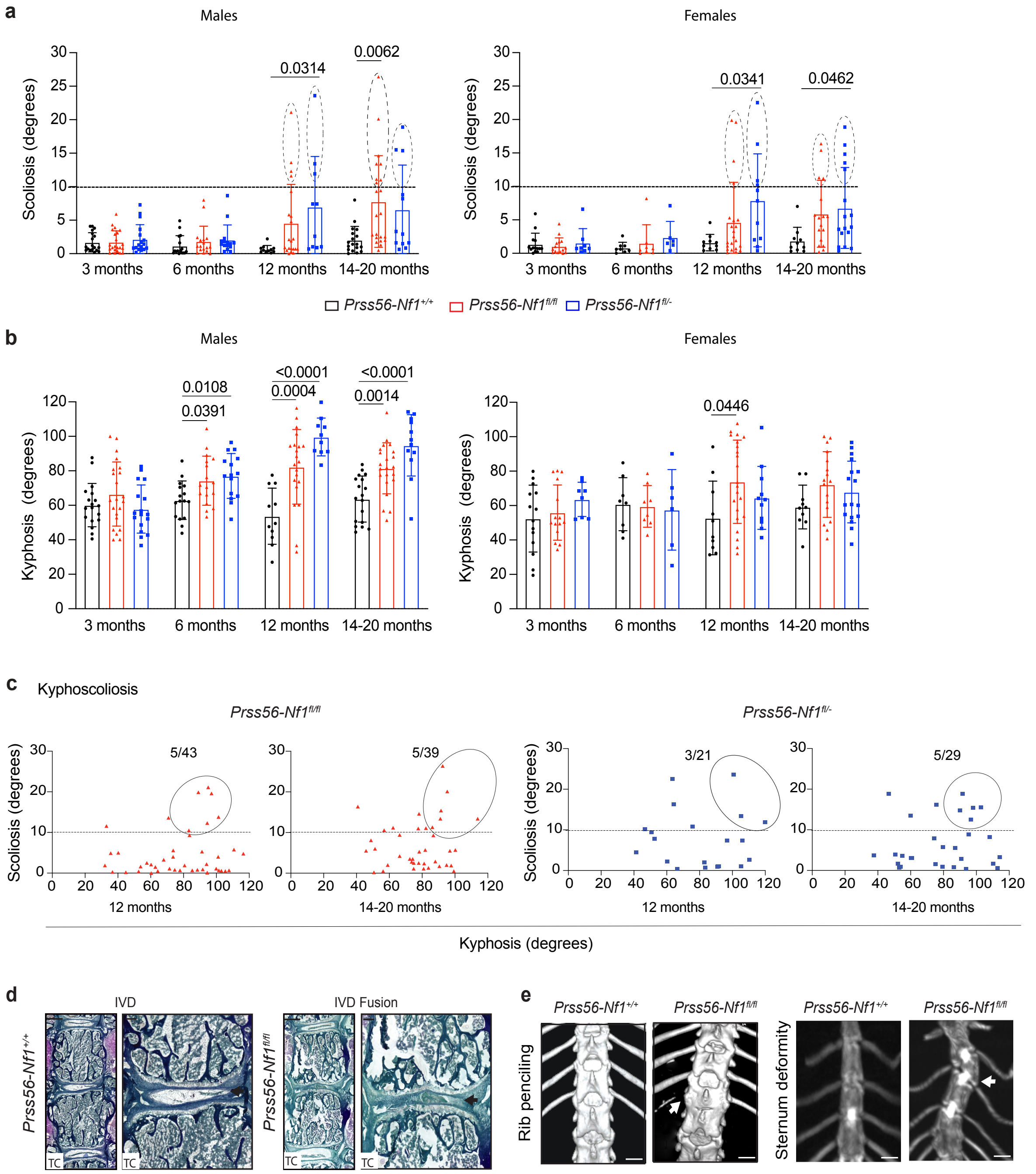

a. Analyses of scoliosis angle in male and female mice at 3-, 6-, 12-, and 14–20 months of age. Scoliosis was observed in both male and female *Prss56-Nf1*$^{fl/fl}$ and *Prss56-Nf1*$^{fl/-}$ mutant mice (dotted circles) from 12 months but not in *Prss56-Nf1*$^{+/+}$ control mice (n=6-22 mice/group) **b.** Analyses of kyphosis angle in male and female mice at 3-, 6-, 12-, and 14-20 months of age. Significant increase of kyphosis was observed in males *Prss56-Nf1*$^{fl/fl}$ and *Prss56-Nf1*$^{fl/-}$ mutant mice from 6 months of age compared to *Prss56-Nf1*$^{+/+}$ control mice (n=6-22 mice/group). Data are presented as mean ± SD. Statistical significance was determined using one-way ANOVA followed by Tukey's multiple comparisons test. $p < 0.05$ was considered statistically significant. **c.** Graphs showing kyphoscoliosis (circles) in 11.62% (5/43) and 12.82% (5/39) of *Prss56-Nf1*$^{fl/fl}$ and 14.28% (3/21) and 17.24% (5/29) of *Prss56-Nf1*$^{fl/-}$ mutant mice at 12 and 14-20 months of age respectively. **d.** Vertebrae stained with Trichrome (TC) illustrating normal intervertebral disc in control mice left panel (black arrow) and intervertebral disc fusion in mutant right panel (black arrow) at 14-20 months of age. Scale bars: low magnification 200 µm, high magnification 100 µm **e.** Micro-CT images illustrating rib penciling and sternum deformity (white arrows) in mutant mice compared to control mice at 14-20 months of age. Scale bar 1 mm.

Supplemental Figure 3: Prss56-derived Tdtom+ cells localized in the vertebrae are significantly increased starting from the embryonic stage until adulthood in *Prss56-Nf1* KO mice

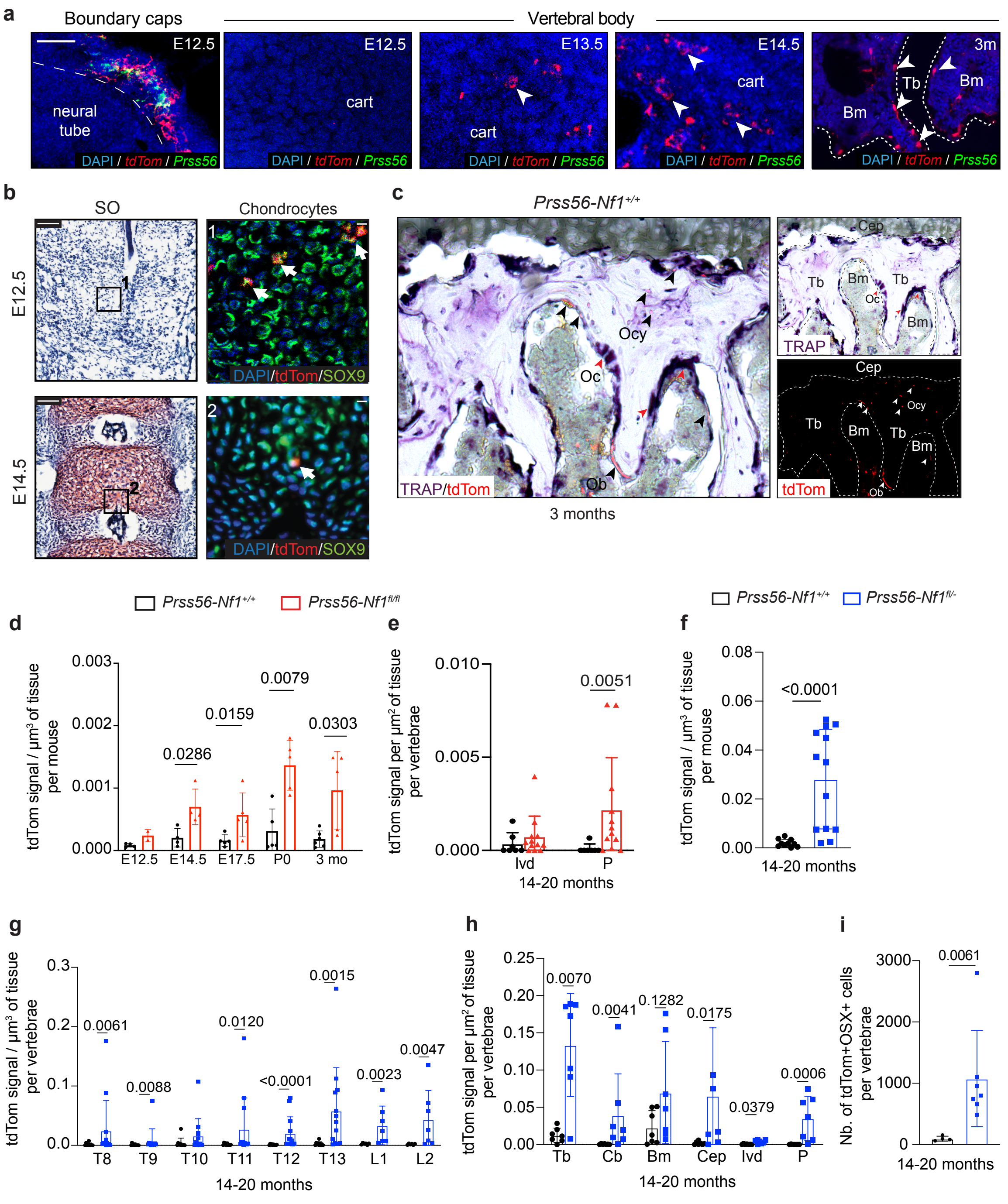

a. RNAscope experiment using *tdTom* and *Prss56* probes on boundary caps and vertebrae body from embryonal stages E12.5, E13.5, and E14.5 and 3 months of age in *Prss56-Nf1*$^{+/+}$ control mice. Images illustrating cells expressing *tdTom* and *Prss56* at E12.5 in boundary caps and cells expressing *tdTom* but not *Prss56* in the cartilaginous matrix (cart) of the vertebral body at embryonal stages E13.5, and E14.5 and lining trabecular bone (Tb) at 3 months of age (n=3 samples/time point). Bm=Bone marrow Scale bar 50 µm **b.** Left: Vertebrae of *Prss56-Nf1*$^{+/+}$ control mice stained with SafraninO (SO) at embryonic stages E12.5 and E14.5. Right: high magnification of immunofluorescence illustrating the presence of Prss56-derived SOX9+ chondrocytes (white arrows). Scale bar: low magnification on left 100 µm, high magnification on right 10 µm. **c.** Left: Overlay of a magnified vertebra stained with TRAP, imaged in bright field and fluorescence channel for tdTom+ cells, showing no TRAP+tdTom+ cells. Right: Individual views of the same vertebrae region, with the bright field image (up) and the fluorescence channel image (down) shown separately. Tb=trabecular bone, Bm=Bone marrow, Cep=cartilage endplate, Ob=osteoblasts, Ocy=osteocytes (black arrowheads)

Oc=osteoclasts (red arrowheads) (n=3 samples) **d.** Quantification of tdTom signal in vertebrae showing a significant increase of tdTom signal/volume of tissue per mouse in *Prss56-Nf1*$^{fl/fl}$ mutant compared to *Prss56-Nf1*$^{+/+}$ control mice at embryonic stages E14.5, E17.5 and day 0 of birth (P0) until 3 months of age (n=4-5 mice/group). **e.** Quantification of tdTom signal/surface of tissue in intervertebral disc (Ivd) and periosteum (P), showing a significant increase in the periosteum of *Prss56-Nf1*$^{fl/fl}$ mutant compared to *Prss56-Nf1*$^{+/+}$ control mice (n=7-11 vertebra/group). **f.** Quantification of tdTom signal in vertebrae showing significant increase of tdTom signal/volume of tissue per mouse in 14-20 month-old *Prss56-Nf1*$^{fl/-}$ mutant compared to *Prss56-Nf1*$^{+/+}$ control mice (n=10-13 mice/group). **g.** Quantification of tdTom signal/volume of tissue in individual vertebrae from T8-L2 region showing a significant increase in *Prss56-Nf1*$^{fl/-}$ mutant compared to *Prss56-Nf1*$^{+/+}$ control mice at 14-20 months of age and more specifically in the lower thoracic-lumbar region (T11-L2) (n=10-13 vertebra/group). **h.** Quantification of tdTom signal/surface of tissue in trabecular bone (Tb), cortical bone (Cb), bone marrow (Bm), cartilage endplate (Cep), intervertebral disc (Ivd) and periosteum (P) in the vertebrae showing significant increase in *Prss56-Nf1*$^{fl/-}$ mutant compared to *Prss56-Nf1*$^{+/+}$ mice in Tb, Cb, Bm, Cep, Ivd and P (n=7 vertebra/group). **i.** Quantification of the number of tdTom+OSX+ cells in vertebrae showing a significant increase in *Prss56-Nf1*$^{fl/-}$ mutant compared to *Prss56-Nf1*$^{+/+}$ control mice at 14-20 months of age (n=4-7 vertebra/group). Data are presented as mean ± SD. Statistical significance was determined using Mann–Whitney U test. $p < 0.05$ was considered statistically significant.

Supplemental Figure 4: Identification and distribution per sample of tdTom+ cells in the spine of control and *Prss56-Nf1* KO mice by scRNAseq analyses

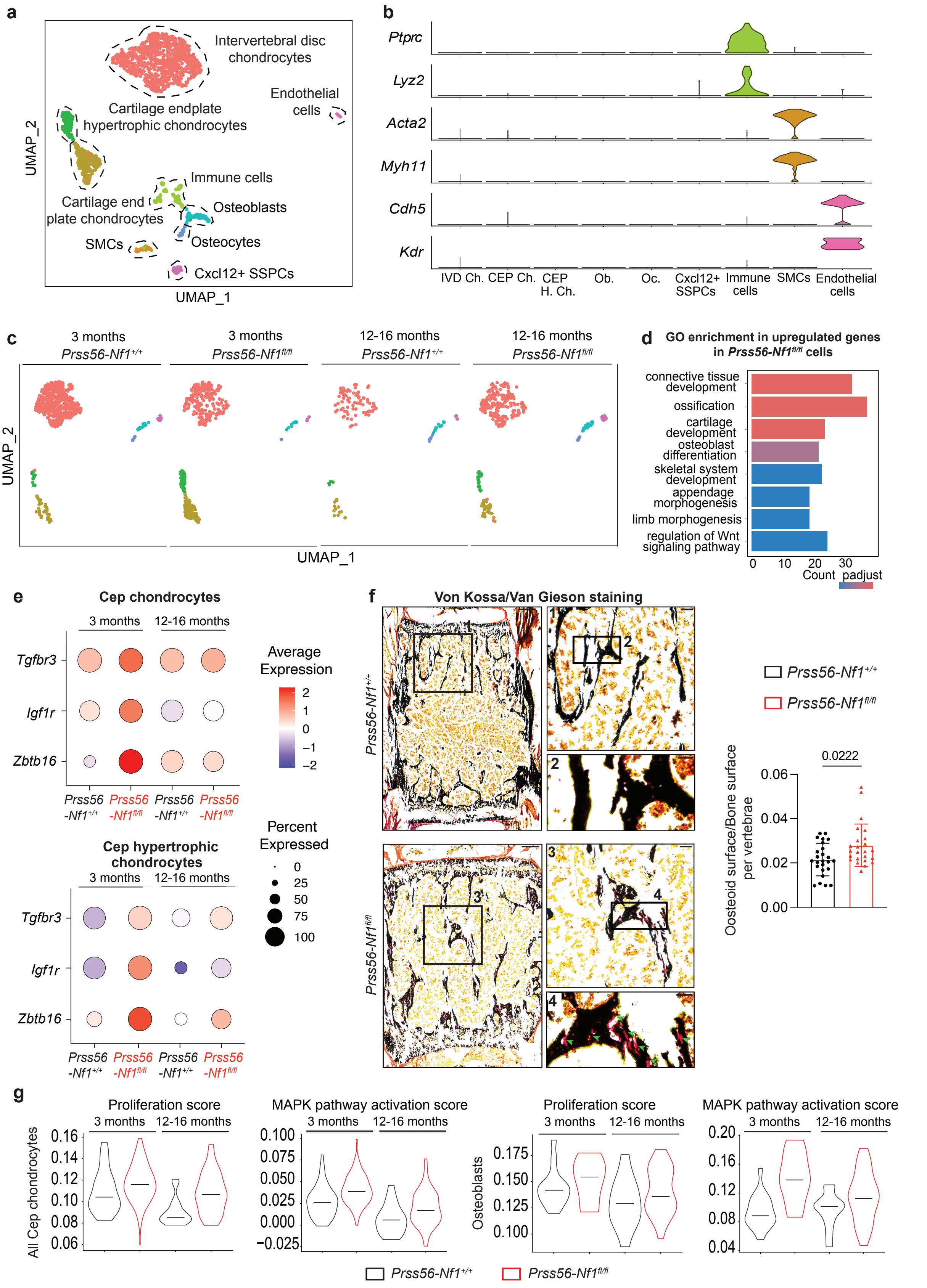

a. UMAP projection of color-coded clustering of the integration of 3 and 12-16-month-old *Prss56-Nf1*$^{+/+}$ control and *Prss56-Nf1*$^{fl/fl}$ mutant mice datasets before removal of hematopoietic, smooth muscle and endothelial cells. Each population was identified and delimited by black dashed lines. **b.** Violin plots of key marker genes

of the immune, smooth muscle cells (SMCs) and endothelial cell populations. IVD (intervertebral disc), Cep (cartilage endplate), Cep.H (Cartilage endplate hypertophic chondrocytes), Ob (osteoblasts), Oc (Osteoclasts), SSPCs (Skeletal stem progenitor cells). **c.** UMAP projection of color-coded clustering of *Prss56-Nf1*$^{fl/fl}$ mutant and *Prss56-Nf1*$^{+/+}$ control samples at 3 and 12-16 months of age separately. **d.** Gene ontology analyses of upregulated genes in *Prss56-Nf1*$^{fl/fl}$ mutant cells. **e.** Dot plot of the expression of osteogenic genes (*Tgfbr3*, *Igf1r*, *Zbtb16*) in (Cep) cartilage endplate chondrocytes (up) and hypertrophic cartilage endplate chondrocytes (down). **f.** Left: Longitudinal sections of the vertebrae from 16-month-old *Prss56-Nf1*$^{+/+}$ control (up) and *Prss56-Nf1*$^{fl/fl}$ mutant (down) mice stained with Von Kossa/Von Gieson, illustrating the presence of osteoid surface within vertebrae. High magnification corresponds to the black box area and shows pink non mineralized tissue (green arrowheads) (box 3-4) compared to control vertebrae mineralized (box 1-2). Scale bar: big boxes (low magnification), 200 µm; small boxes (high magnification) 1 and 3, 100 µm; and boxes 2 and 4, 25 µm. Right: Quantification of osteoid surface per bone surface in vertebrae showing significant increase of osteoid surface per vertebrae in 14–20-month-old *Prss56-Nf1*$^{fl/fl}$ mutant compared to *Prss56-Nf1*$^{+/+}$ control mice (n=24-25 vertebra/group). Data are presented as mean ± SD. Statistical significance was determined using Mann–Whitney U test. $p < 0.05$ was considered statistically significant. **g.** Violin plots of the proliferation score and MAPK pathway activation score in all cartilage endplate chondrocytes (hypertrophic and non-hypertrophic) and osteoblasts in *Prss56-Nf1*$^{fl/fl}$ mutant and *Prss56-Nf1*$^{+/+}$ control mice at 3 and 12-16 months of age.

Supplemental Figure 5: Changes in vertebral bone parameters of *Prss56-Nf1* KO mice correlate with the increase in kyphosis spine curvature

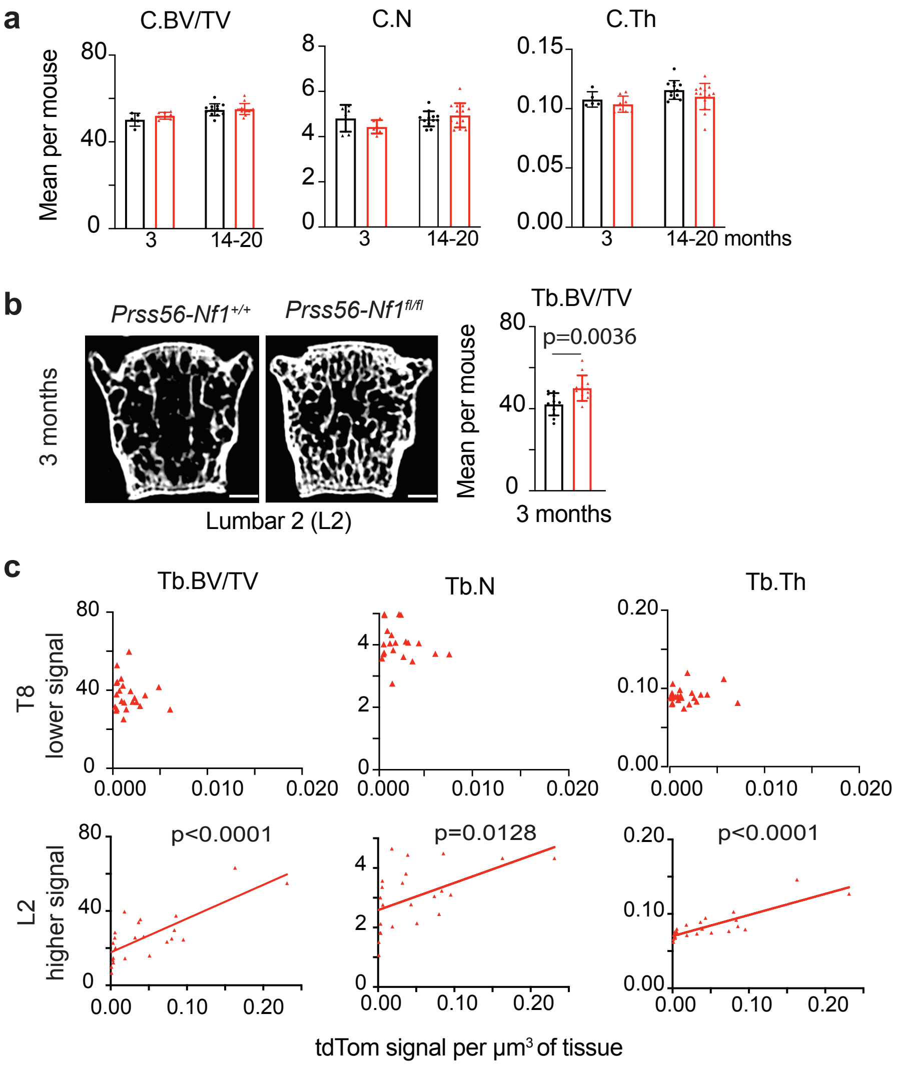

a. Ex-vivo high-resolution analyses per mouse of cortical bone volume/total volume (C. BV/TV), cortical number (C.N), cortical thickness (C.Th) in the vertebrae showing no significant differences in *Prss56-Nf1$^{fl/fl}$* mutant mice compared to *Prss56-Nf1$^{+/+}$* control mouse at 3 and 14-20 months of age (n=5-14 mice/group) **b.** Left: Ex-vivo high-resolution micro-CT images in L2 vertebrae, illustrating more trabecular bone (Tb) in *Prss56-Nf1$^{fl/fl}$* mutant compared to *Prss56-Nf1$^{+/+}$* control mice at 3 months of age. Scale bar 0.1 mm. Right: Ex-vivo high-resolution analyses per mouse showing increased trabecular bone volume/total volume (Tb. BV/TV) in *Prss56-Nf1$^{fl/fl}$* mutant mice compared to *Prss56-Nf1$^{+/+}$* control mice at 3 months of age (n=11-12 mice/group) Data are presented as mean ± SD. Statistical significance was determined using Mann–Whitney U test. $p < 0.05$ was considered statistically significant. **c.** Correlation analyses performed between trabecular bone volume/total volume (Tb. BV/TV), trabecular number (Tb.N), trabecular thickness (Tb.Th) and tdTom signal/tissue volume in individual vertebrae showing no correlation in thoracic 8 (T8) vertebrae with low tdTom signal (upper correlation graphs) and positive correlation in lumbar 2 (L2) vertebrae with high tdTom signal (lower correlation graphs) in *Prss56-Nf1$^{fl/fl}$* mutant mice at 14-20 months of age (n=23 vertebra) Correlation was evaluated using Pearson's correlation coefficient.

Supplemental Figure 6: Absence of correlation between the presence of paraspinal plexiform neurofibromas and spine deformities in *Prss56-Nf1* KO mice

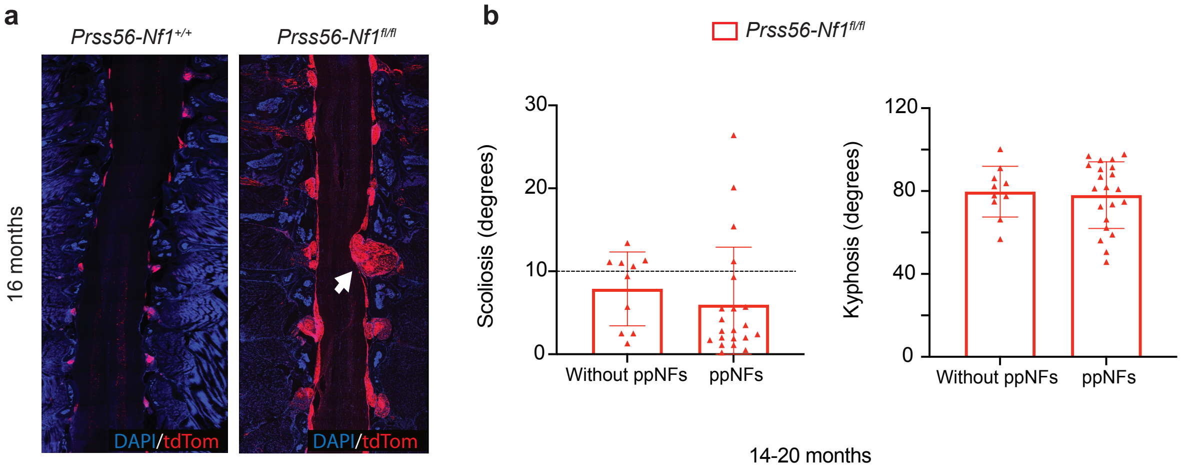

a. Longitudinal sections stained with DAPI of the spinal cord showing no paraspinal plexiform neurofibromas (ppNFs) in *Prss56-Nf1*$^{+/+}$ control mice and the presence of ppNFs (white arrow) in *Prss56-Nf1*$^{fl/fl}$ mutant mice. **b.** Analyses of kyphosis and scoliosis angles in *Prss56-Nf1*$^{fl/fl}$ mutant mice showing no significant difference between mice with and without ppNFs at 14-20 months of age (n=10-22 mice/group) . Data are presented as mean ± SD. Statistical significance was determined using Mann-Whitney U test. $p < 0.05$ was considered statistically significant.

Supplemental Figure 7: Individual mice follow up in *Prss56-Nf1$^{fl/-}$* mutant group treated with vehicle or combined inhibitors Selumetinib + RMC-4550.

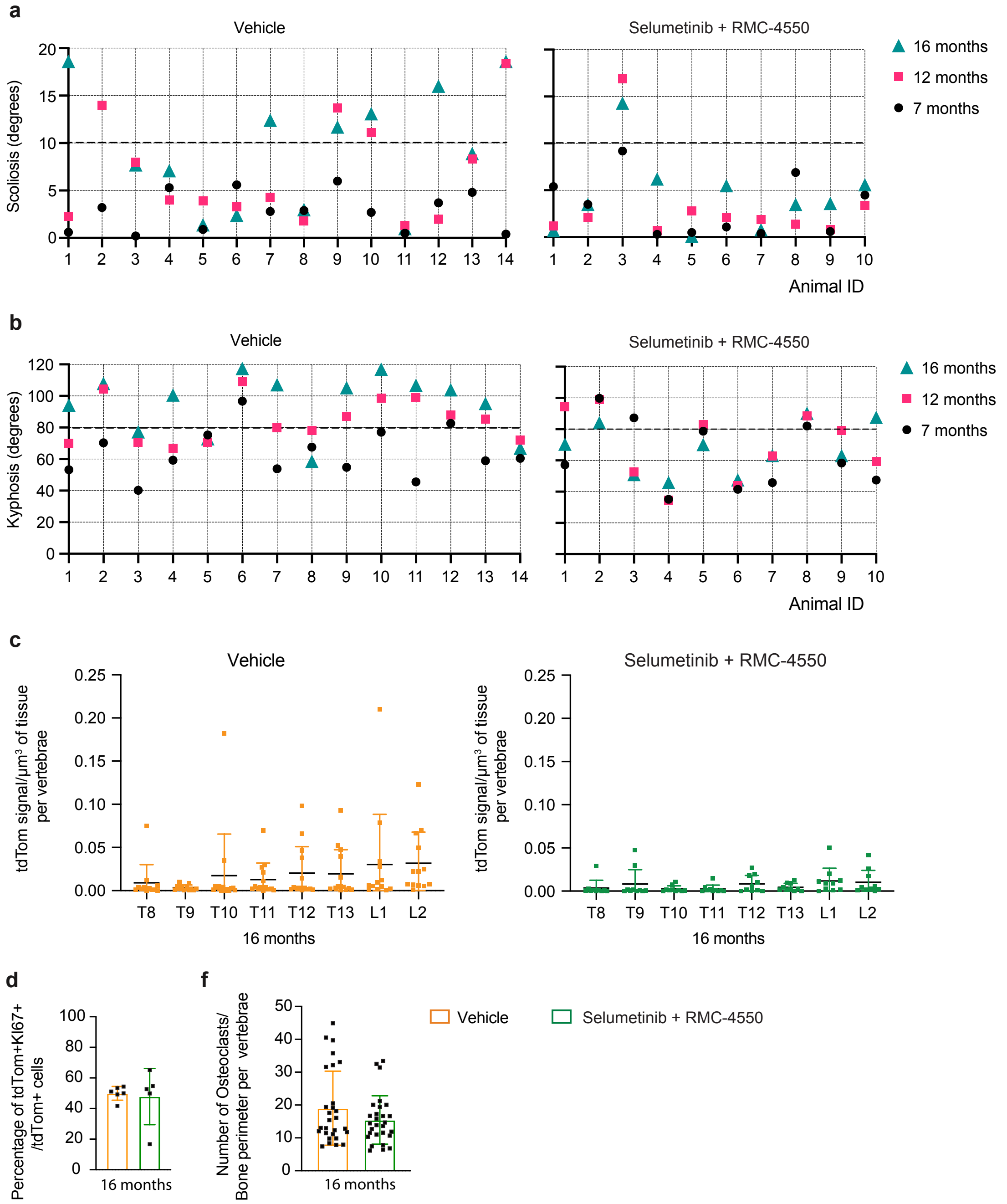

a. Analyses of scoliosis angle overtime in individual mice from *Prss56-Nf1$^{fl/-}$* mutant group treated with vehicle or Selumetinib+RMC-4550. Scoliosis was defined as a lateral curvature >10°. Each animal ID present one mouse evaluated at 7-, 12-, and 16 months of age. Scoliosis is present in vehicle treated mice beginning at 12 months of age but not Selumetinib+RMC-4550 treated mutant mice except in one mouse (n=10-14 mice/group). **b.** Analyses of kyphosis angle overtime in individual mutant mice treated with vehicle or Selumetinib+RMC-4550. Each animal ID present one mouse evaluated at 7-, 12-, and 16 months of age. Kyphosis angle >80° is frequently present in vehicle treated mice beginning at 12 months of age but not in Selumetinib+RMC-4550 treated mutant mice (n=10-14 mice/group). **c.** Quantification of tdTom signal/volume of tissue in individual vertebrae from T8-L2 region showing more tdTom signal in the lower thoracic-lumbar region (T11-L2) in vehicle treated (left) compared to Selumetinib+RMC-4550 treated mutant mice (right) (n=9-14 vertebra/group). **d.** Quantification of the percentage of tdTom+Ki67+ cells showing no difference in the vertebrae of Selumetinib+RMC-4550 treated and vehicle treated mutant mice (n=5-6 vertebra/group). **f.** Quantification of the number of osteoclasts/bone perimeter in vertebrae showing no significant difference in Selumetinib+RMC-4550 treated and to vehicle treated mutant mice (n=28 vertebra/group). Data are presented as mean ± SD. Statistical significance was determined using Mann–Whitney U test. $p < 0.05$ was considered statistically significant.

Supplemental Table 1: Detailed information on the frequency of vertebral anomalies detected in *Prss56Nf1*-KO mutant mice (related to Figure 1)

Genotype	Vertebral fusion		Vertebral wedging		Vertebral scalloping		Rib penciling		Sternum fusion	
	3 months	14-20 months	3 months	14-20 months	3 months	14-20 months	3 months	14-20 months	3 months	14-20 months
Prss56-Nf1$^{+/+}$	0/30 (0%)	0/24 (0%)	0/30 (0%)	0/24 (0%)	0/30 (0%)	0/24 (0%)	0/30 (0%)	0/24 (0%)	0/30 (0%)	0/24 (0%)
Prss56-Nf1$^{fl/fl}$	3/29 (10.3%)	2/32 (6.25%)	na	1/19 (5.26%)	na	2/19 (10.52%)	0/29 (0%)	2/32 (6.25%)	na	4/32 (12.5%)
Prss56-Nf1$^{fl/-}$	5/27 (18.5%)	3/23 (13.04%)	na	na	na	na	0/27 (0%)	1/26 (3.8%)	na	1/16 (6.25%)

na* not analysed

No vertebral anomalies were detected in *Prss56-Nf1*$^{+/+}$ control mice analyzed at 3 and 14-20 months of age. Vertebral fusion, wedging, scalloping, rib penciling, and sternum deformity were detected in different percentages (3%-18%) in *Prss56-Nf1*$^{fl/fl}$ and *Prss56-Nf1*$^{fl/-}$ mutant mice analyzed at 3 and 14-20 months of age. na* not analyzed
